# Markers of environmental enteric dysfunction are associated with changes in pharmacokinetics of praziquantel in preschool age children with *Schistosoma mansoni* infection in Albertine region of Uganda

**DOI:** 10.64898/2026.09.02.26362031

**Published:** 2026-09-07

**Authors:** Andrew Edielu, Bonniface Obura, Patrice A. Mawa, Martin J. Holland, Emily L. Webb, Alison M. Elliott, Gloria Kakoba Ayebazibwe, Hannah Wei Wu, Nick Mancini, Fabian C. Fischer, Susannah Colt, Meagan A. Barry, William Hope, Catriona Waitt, Jennifer F. Friedman, Amaya L. Bustinduy

## Abstract

**Introduction:**

Praziquantel (PZQ) is the only widely available chemotherapy that is effective against all species of schistosomes. Environmental enteric dysfunction (EED) is an acquired intestinal disorder of altered gut function whose effect on drug pharmacokinetics has not been directly explored.

**Methods:**

Preschool-age children infected with *S. mansoni* were randomized to receive 40mg/kg or 80mg/kg of crushed PZQ tablets. Plasma PZQ concentrations were quantified using ultra-high performance liquid chromatography mass spectrometry. Maximum concentration (C _max_ ), time to C _max_ (T _max_ ) and area under the curve (AUC) of PZQ were calculated. Biomarkers of intestinal inflammation (stool calprotectin), epithelial damage (plasma Intestinal Fatty Acid Binding Protein (IFABP)), permeability (urine lactulose:mannitol (LM) ratio and alpha-1 antitrypsin (AAT)), microbial translocation (plasma Endotoxin core antibodies (EndoCAb), systemic inflammation (plasma C-reactive protein (CRP)), and presence of faecal occult blood (FOB) were measured. Using linear regression, we assessed association of AUC, C _max_ , T _max_ and R- to S-PZQ exposure with each biomarker, adjusting for dose, age, and sex.

**Results:**

Of the 184 participants included in the final analysis, 91 received 40mg/kg and 93 received 80mg/kg of PZQ. The T _max_ was associated with LM ratio (β=0.06, 95% CI 0.02 – 0.11, p=0.003) and calprotectin (β=0.001, 0.0002 – 0.002, p=0.013). CRP was associated with AUC (β=0.13, 95% CI 0.06 – 0.21, p=0.001) and C _max_ (β=0.12, 95% CI 0.05 – 0.21, p=0.002). Calprotectin (β=0.12, 95% CI 0.04 - 0.20, p=0.003) and AAT (β=0.09, 95% CI 0.02 – 0.15, p=0.008) were associated with a higher R-PZQ/S-PZQ AUC ratio, while CRP was not (p=0.38).

**Conclusion:**

Elevated intestinal inflammatory markers were associated with increased T _max_ , indicating reduced rate of absorption and relative increase in exposure to the active R-enantiomer. Systemic inflammation was associated with higher C _max_ and AUC, implying increased exposure to PZQ. This is the first report linking alterations in praziquantel pharmacokinetics to EED markers and systemic inflammation.

**Author summary:**

Schistosomiasis, also known as bilharzia, affects approximately 240 million people in 79 countries with children in Africa bearing almost 50% of the burden. Praziquantel (PZQ), an oral drug, is the only widely available treatment for the disease. However, some studies have reported differences in pharmacokinetics and efficacy of PZQ across geographical locations suggesting environmental factors to affect drug response. We hypothesized that environmental enteric dysfunction, a disorder of altered intestinal function, may affect absorption and metabolism of PZQ because of the damage it inflicts on the intestinal mucosa, and subsequent systemic inflammation due to leakage of intestinal contents, a process referred to as “microbial translocation”. Our findings indicate that markers of intestinal inflammation were associated with reduced rate of absorption while systemic inflammation was associated with increased plasma concentrations and overall exposure to PZQ. The reduced rate of absorption may result from destruction of the intestinal absorptive surface while increased plasma concentration and exposure may be due to reduced activity of PZQ-metabolizing enzymes in the liver. These observations could inform approaches for optimizing the effectiveness of PZQ by accounting for inflammation. This has implications for the use of PZQ as an effective control strategy and subsequent elimination of schistosomiasis by 2030, in line with World Health Organization targets.

## Full Text Availability

The license terms selected by the author(s) for this preprint version do not permit archiving in PMC. The full text is available from the preprint server.

